# A156 MUCOSA-ASSOCIATED MICROBIOTA OF ILEOCOLONIC CROHN’S DISEASE PATIENTS IS DISTINCT FROM COLONIC CROHN’S DISEASE AND ULCERATIVE COLITIS PATIENTS INDEPENDENT OF BIOPSY SITE, ENDOSCOPIC INFLAMMATION AND HOST GENETICS

**DOI:** 10.1093/jcag/gwab049.155

**Published:** 2022-02-21

**Authors:** C Hernandez-Rocha, S Nayeri, W Turpin, K Borowski, J Stempak, M S Silverberg

**Affiliations:** Zane Cohen Centre for Digestive Diseases, Lunenfeld-Tanenbaum Research Institute, Sinai Health System, Toronto, ON, Canada

## Abstract

**Background:**

Colonic IBD encompassing ulcerative colitis (UC) and isolated colonic Crohn’s disease (cCD) shows significant clinical, therapeutic response and genetic differences compared to ileocolonic CD (icCD). Elucidating the microbial signatures characterizing these subphenotypes could help to understand the causal factors underlying these clinical dissimilarities

**Aims:**

We compared the mucosal microbial diversity and differential abundance (DA) among disease locations (UC, cCD and icCD) accounting for potential clinical, endoscopic, and genetic confounders

**Methods:**

Healthy control (HC), UC, cCD and icCD patients (including ileal and ileocolonic involvement) underwent colonoscopy. Biopsy samples were obtained from terminal ileum (TI), ascending colon (AC) and sigmoid colon (SC) for 16s rRNA gene profiling. Patients with prior ileocecal resection, IBD-unclassified and antibiotic exposure within 3 months before colonoscopy were excluded. Endoscopic inflammation was defined as a segmental Mayo endoscopic subscore = 0 in UC and a simple endoscopic score ≤ 2 in CD. A blood sample was drawn for genotyping and a weighted genetic risk score (GRS) was built based on 169 IBD risk variants found in our cohort. Alpha diversity (Chao1) and DA between IBD subphenotypes were compared using a linear mixed-effects model with subjects as random effect and adjusted for biopsy site, endoscopic inflammation, age, sex, and GRS. For DA analysis, the MaAsLin2 protocol was applied. All p-values were corrected by false discovery rate (FDR) with < 0.05 considered significant

**Results:**

A total of 199 IBD patients and 44 HC with a mean age of 37.2 ± 14 were recruited. Of these, 113 (46.5%) were female. At colonoscopy, 535 biopsy samples (TI = 178, AC = 123 and SC = 234) were obtained. Considering disease location, 254, 55 and 148 samples were obtained from UC, cCD and icCD patients, respectively. A total of 168 samples (31.4%) showed endoscopic inflammation. Alpha diversity was significantly reduced in icCD when compared to either HC, UC or cCD. MaAsLin2 identified that the genera *Agathobacter* and *Faecalibacterium*, as well as the family Ruminococacceae and the order Oscillospirales were significantly reduced in icCD when compared to either HC, UC or cCD. These findings were independent of age, sex, endoscopic inflammation, biopsied site, and GRS. UC and cCD did not show differences in their microbial profile

**Conclusions:**

Mucosal samples from UC and cCD patients showed marked similarities in their microbial profile while icCD is characterized by a significant decrease in diversity and beneficial microbes. These data suggest that disease location is the main driver of the mucosal microbial landscape independent of IBD GRS

**Funding Agencies:**

NoneNIDDK IBD Genetics Consortium

